# Knockout of the *TauT* Gene Predisposes C57BL/6 Mice to Streptozotocin-Induced Diabetic Nephropathy

**DOI:** 10.1371/journal.pone.0117718

**Published:** 2015-01-28

**Authors:** Xiaobin Han, Andrea B. Patters, Takashi Ito, Junichi Azuma, Stephen W. Schaffer, Russell W. Chesney

**Affiliations:** 1 Department of Pediatrics, University of Tennessee Health Science Center, and the Children’s Foundation Research Institute at Le Bonheur Children’s Hospital, Memphis, TN, United States of America; 2 Department of Pharmacy, College of Pharmacy, Hyogo University, Kobe, Japan; 3 Department of Pharmacology, University of South Alabama, Mobile, AL, United States of America; Children’s Hospital Boston/Harvard Medical School, UNITED STATES

## Abstract

Diabetic nephropathy is the leading cause of end stage renal disease in the world. Although tremendous efforts have been made, scientists have yet to identify an ideal animal model that can reproduce the characteristics of human diabetic nephropathy. In this study, we hypothesize that taurine insufficiency is a critical risk factor for development of diabetic nephropathy associated with diabetes mellitus. This hypothesis was tested *in vivo* in *TauT* heterozygous (*TauT* ^+/-^) and homozygous (*TauT^-/-^*) knockout in C57BL/6 background mice. We have shown that alteration of the *TauT* gene (also known as SLC6A6) has a substantial effect on the susceptibility to development of extensive diabetic kidney disease in both *TauT* ^+/-^ and *TauT^-/-^*mouse models of diabetes. These animals developed histological changes characteristic of human diabetic nephropathy that included glomerulosclerosis, nodular lesions, arteriosclerosis, arteriolar dilation, and tubulointerstitial fibrosis. Immunohistochemical staining of molecular markers of smooth muscle actin, CD34, Ki67 and collagen IV further confirmed these observations. Our results demonstrated that both homozygous and heterozygous *TauT* gene deletion predispose C57BL/6 mice to develop end-stage diabetic kidney disease, which closely replicates the pathological features of diabetic nephropathy in human diabetic patients.

## Introduction

The impact of diabetic kidney disease on the health of the United States population is staggering. Diabetes and hypertension, highly prevalent in older patients, are major risk factors for chronic kidney disease and end stage renal disease [1]. Hence, this form of chronic kidney disease poses a considerable medical and public health challenge, particularly in the elderly. However, the marked variability in the development of chronic kidney disease in individuals with hypertension and diabetes suggests that additional underlying factors contribute to its development. While clear progress has been made in understanding the disease process, there has been limited success in identifying specific factors that cause or even predict human nephropathy and its progression. One of the reasons for the slow evolution in the understanding of diabetic nephropathy is the lack of reliable animal models that mimic human disease.

More than three decades ago it was observed that intensive diabetes therapy in humans with the aim of normalizing glucose control could reduce both the development and progression of diabetic retinopathy, nephropathy and cardiovascular disease [2]. The value of intensive treatment on the complications of diabetes ushered in an era of stricter glucose control [3] Diabetic nephropathy, as a component of diabetic vascular disease, worsens most diabetic complications. In particular, the risk of morbidity and mortality from cardiovascular disease is increased several fold [4]. Cardiac left ventricular function is also impaired in both insulin-dependent and in non-insulin-dependent diabetic patients [1]. Hence, the impact of sustained diabetes is considerable.

In 2001, the Animal Models of Diabetic Complications Consortium was initiated by the National Institutes of Health to develop animal models for enhancing effective therapies and preventive strategies for diabetic nephropathy. Though some progress has been made in model development, mouse phenotyping, strain analysis, and the understanding of pathogenesis of diabetic complications [5–9], the primary goal of the consortium has not yet been attained.

Recent studies show that both BRKO-Akita and BTBR *ob/ob* mice develop more advanced diabetic nephropathy than other murine models [10,11]. However, both of these mouse models have certain limitations, especially with regard to end stage renal disease. These animals fail to develop severe progressive renal insufficiency, specifically characterized as >50% decline in glomerular filtration rate (GFR) during the lifetime of the animal. Additionally, the BTBR *ob/ob* mouse model is dependent on leptin deficiency, which is not a characteristic of human diabetes [10].The lack of reliable animal models that closely mimic human disease has delayed the identification of specific factors that cause diabetic nephropathy. This may be largely because certain key risk factor(s) in diabetes affect humans and rodents differently. Metabolically healthy obesity (MHO) is a major risk factor for type 2 diabetes and cardio-vascular disease, and controlling MHO benefits morbidly obese individuals [12]. Diabetic kidney disease is a major result of diabetic complications, which is strongly associated with MHO [13,14]. Therefore, finding an ideal animal model of diabetic kidney disease could help us to better understanding the insight of the disease during diabetes. In light of the beneficial role of taurine in ameliorating metabolic disease by reducing blood glucose levels in diabetic animals [15], and its protective role in cisplatin-induced acute kidney injury [16], we proposed that taurine may help protect kidneys from disease.

The goal of this study was to determine whether the taurine deficiency observed in diabetic patients [17,18] constitutes a potential key risk factor for chronic kidney disease by contributing to diabetic nephropathy. We posit that an animal model that displays the clinical features of diabetes mellitus and taurine deficiency as the result of *TauT* deficiency might faithfully mimic human diabetic nephropathy.

## Methods

### Mice

Inbred male *TauT* ^–/—^and age-matched male *TauT* ^+/+^ (wild-type) littermate mice (C57BL/6) were produced by mating *TauT* ^+/—^inbred male and female breeders and then genotyped as described [19]. Six groups of mice were examined. Twenty of each of the three genotypes (wild-type, *TauT* ^+/–^, and *TauT* ^–/–^) were randomly divided into two groups (n = 10).

### Treatment

Mice (7–8 weeks of age) were fasted for 4 h, then briefly anaesthetized with isoflurane and injected intraperitoneally with 50 mg/kg STZ (made fresh in 0.05 M sodium citrate buffer, pH 4.5) for five consecutive days [8]. Another group of animals of each genotype (n = 10) received intraperitoneal injections of citrate buffer alone as control. Development of diabetes was defined by blood glucose >250 mg/dL and verified one week after the first STZ injection. Animals were visually monitored at regular intervals (2–3 times weekly). Criteria for intervention were weight loss, dehydration, cataracts, lethargy, and diabetic coma. Diabetic mice with suspected problems were examined every day, including measurements of food and water intake. Appropriate treatment or humane euthanasia was applied based on the recommendations of veterinarians in the Department of Laboratory Animal Medicine at the University of Tennessee Health Science Center. Liquid nutrition supplements were used to prevent weight loss in severely diabetic animals when it was needed. Mice with non-fasting blood glucose levels above 35 mmol/L or severe dehydration (weight loss more than 20% during the course of diabetic nephropathy) were determined to have reached the endpoint of the study and were euthanized by an overdose of isoflurane and cervical dislocation. In the present study, five of 10 diabetic mice met these criteria and were euthanized using the method described above. The study was ended after hyperglycemia was established for at least 20 weeks. Mice were sacrificed by overdose of inhaled isoflurane followed by cervical dislocation. Blood urea nitrogen (BUN) was measured by using a BUN assay kit (Diagnostic Chemicals Ltd., Oxford, CT). Albumin was measured by using a Mouse Urinary Albumin Detection Kit (Chondrex, Redmond, WA). Creatinine was measured with the Liquid Creatinine Assay (Bioquant, San Diego, CA). Diabetic nephropathy in mice was evaluated using the methods and validation criteria recommended by the Animal Models of Diabetic Complications Consortium (AMDCC; http://www.amdcc.org).

### Ethics Statement

All experiments were performed following a protocol [#12–027.0-C (1533—Mouse)] specifically approved for this entire study by the Animal Care and Use Committee of the University of Tennessee Health Science Center.

### Renal Histology

Kidneys were fixed in 10% formalin neutral buffered solution (Sigma, St. Louis, MO) and embedded in paraffin. Two-micrometer thick sections were stained with the periodic acid-Schiff (PAS) reagent or hematoxylin and eosin (H&E), performed by AML Laboratories (Mariland, MA). Immunohistochemistry was performed by using an Ultra-Sensitive ABC Rabbit IgG Staining Kit following the manufacturer’s instructions (Thermo Scientific, Rockford, IL). Briefly, samples were rehydrated in decreasing ethanol series (100%, 95%, and 70%) for 5 min each. Samples were immersed in 1x PBS (phosphate buffered saline) for 10 min at room temperature, then quenched in a hydrogen peroxide solution (3% H_2_O_2_ in methanol) for 5 min, then washed twice in distilled H_2_O for 10 min. Slides were blocked for 30 min with the kit’s blocking buffer. Primary antibodies (antibodies against taurine transporter protein, SMA, CD34, Ki67, or collagen IV purchased from Abcam, Cambridge, MA) were applied to slides and incubated for 1 h. Slides were washed for 10 min with PBS, then a biotinylated secondary antibody was applied and incubated for 1 h. After washing for 10 min with PBS, ABC reagent was applied for 30 min. Finally, immunostaining was detected by using the Metal Enhanced DAB Substrate Kit (Thermo Scientific, Rockford, IL).

For electron microscopy, kidneys were fixed in 10% formalin neutral buffered solution and embedded in epoxy resin, then stained with uranyl acetate and lead citrate. Glomerular basement membrane measurements were performed with JEOL 2000EX transmission electron microscopy with a high-resolution digital camera. The thickness of the GBM was calculated by determining the harmonic mean of a series of orthogonal intercept lengths. At least 35 different segments of GBM per mouse from four mice were measured in each experimental group. The number of podocytes was also counted in these animals at the same time. All quantifications were performed in a blinded manner.

### RNA isolation and real-time PCR

Total RNA was isolated from whole kidney of mouse at 5 months after STZ-induced DM using an RNeasy Mini Kit (Qiagen, Germany). For quantitative real-time RT-PCR, 1.0 µg total RNA isolated from kidneys of all three genotypes of mice was reverse transcribed using an iScript cDNA synthesis kit (Bio-Rad, Hercules, CA, USA) following the manufacturer’s instructions. PCR reactions contained 1 µl of cDNA (equivalent to 50 ng of total RNA), 300 nM each forward (5′-TCGACTTTGTGCTGTCTG-3′) and reverse (5′-GTAGTGGACGACCCTCTT-3′) primers, and 1× iQ SYBR Green supermix (Bio-Rad, Hercules, CA, USA) in a total of 25 µL reaction volume performed with CFX96 Real-Time PCR Detection Systems (Bio-Rad). Relative expression values were evaluated with the 2^-ΔΔ^Ct method using ubiquitin as the housekeeping gene (forward primer: 5′-TGGCTATTAATTATTCGGTCTGCAT-3′, and reverse primer: 5′-GCAAGTGG-CTAGAGTGCAGAGTAA-3′).

### Western blot analysis

Kidney tissue (~10 mg) was transferred into T-PER tissue protein extraction reagent (Thermo Scientific, Rockford, IL) and 1x protease inhibitor cocktail with 1mM phenylmethylsulfonyl fluoride (PMSF; Cell Signaling, Danvers, MA, USA). After three 30-second sonications, samples were centrifuged at 13,000 x *g* for 10 minutes and protein contents in the supernatants were quantified. Samples were stored at -80°C until use. For electrophoresis, samples were prepared by mixing 3x SDS loading buffer (Cell Signaling) with 1x dithiothreitol (DTT). About 50 µg of protein were loaded onto a NuPAGE 4–12% Bis-Tris Gel (Invitrogen, Carlsbad, CA). Proteins were separated at 150 V for 60 minutes and transferred to a nitrocellulose membrane (Invitrogen). Membranes were blocked with Superblock blocking buffer in TBST (Thermo Scientific, Rockford, IL) for 30 minutes and then incubated with primary antibody with gentle agitation overnight at 4°C. After three washes with TBST (15 min once and 5 min twice), the membrane was incubated with secondary antibody in Superblock blocking buffer at room temperature for 1 hour. The membrane was then washed four times (15 min once and 3 x 5 min) and subjected to ECL (Thermo Scientific, Rockford, IL) and analyzed with the FOTO/Analyst Luminary/FX imaging workstation (FOTODYNE INCORPORATED, Hartland, WI).

### Statistical Analysis

We performed all animal experiments twice. The data represent the mean ± standard error of at least four mice. We evaluated differences between two groups by unpaired *t*-test and multiple groups by one-way analysis of variance ANOVA). All computations were performed using GraphPad Prism 5 (GraphPad Software Inc. La Jolla, CA, USA). Significance was defined as *P* < 0.05.

## Results

### TauT Expression and Its Regulation in STZ-induced Diabetic Mice

**Fig 1:**
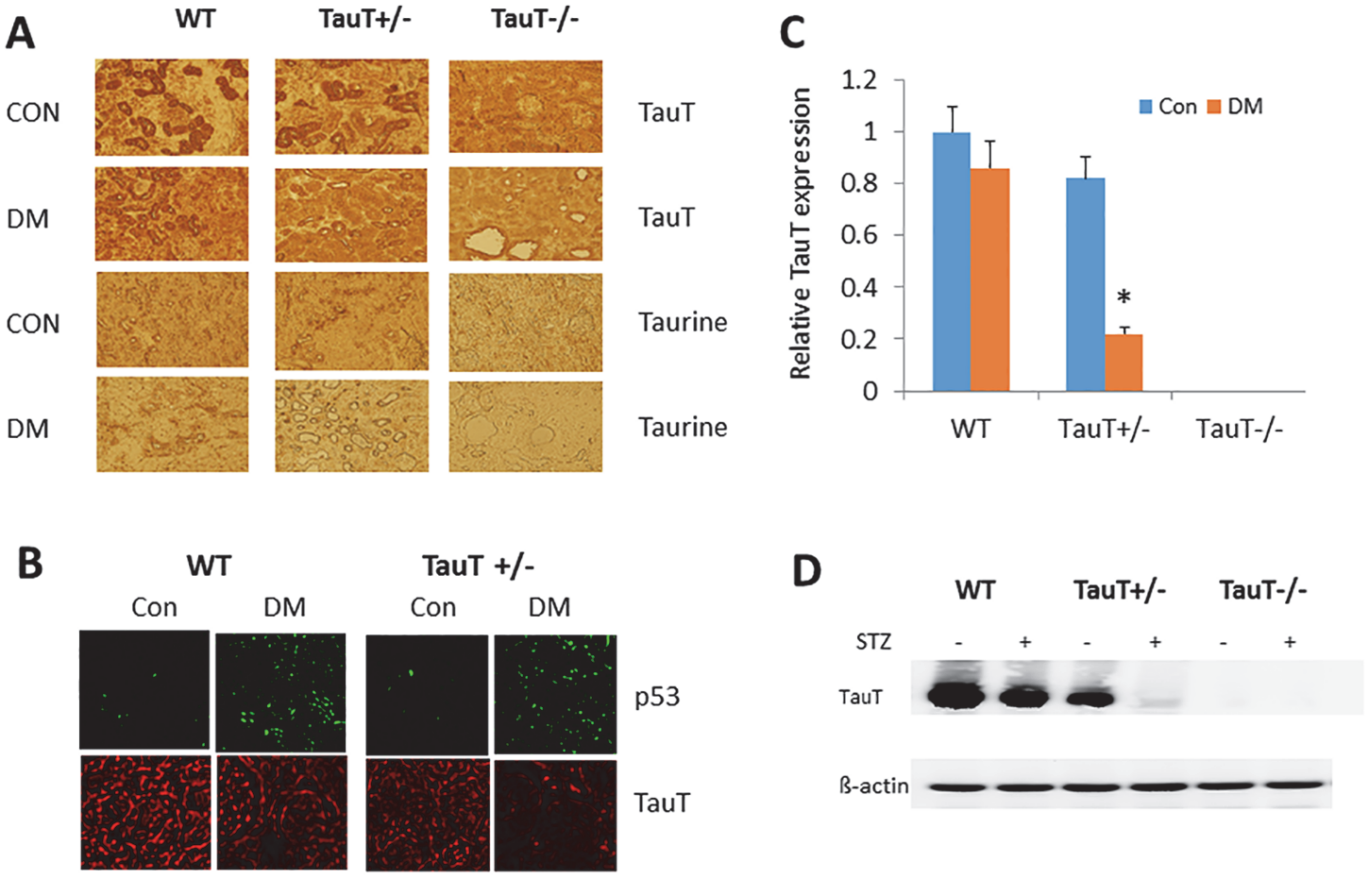
TauT expression and the presence of taurine in kidney tissue of non-diabetic and diabetic wild-type (WT), *TauT*+/-, and *TauT-/-* mice. A) The brown color represents both TauT protein and taurine staining. Con, control, DM, diabetes mellitus. B) Expression of *p53* and *TauT* in both non-diabetic and diabetic wild-type and *TauT* ^+/-^ mice. C) Relative *TauT* expression in both non-diabetic and diabetic wild-type and *TauT* ^-^deficient mice. D) Western blot analysis of TauT protein in the kidneys of both non-diabetic and diabetic wild-type and *TauT* ^-^deficient mice at 5 months of DM. β-actin was used as an internal control for loading of protein samples. *p < 0.05 vs control. Magnification: x200.

We first examined levels of taurine transporter protein (TauT) expression and intracellular taurine using a TauT antibody and a taurine antibody in groups of non-diabetic and diabetic wild-type (WT), *TauT* ^+/-^ and *TauT* ^*-/-*^ mice. As shown in Fig. 1A, high levels of TauT protein were found in the renal tubules of non-diabetic WT mice.

As expected, TauT was not detected in the kidneys of *TauT* ^*-/-*^ mice. Expression of TauT was slightly reduced in diabetic WT mice as compared to non-diabetic WT mice. A relatively high amount of TauT was evident in non-diabetic *TauT* ^+/-^ mice. The amount of TauT was lesser in diabetic *TauT* ^+/-^ mice, and it was not detected in diabetic *TauT* ^*-/-*^ mice. Levels of intracellular taurine matched the levels of TauT in each genotype of mouse, and were highest in non-diabetic WT mice, lower in non-diabetic *TauT* ^+/-^ mice, and absent in non-diabetic *TauT* ^*-/-*^ mice. Because TauT is a specific membrane transporter required for the influx of taurine, intracellular taurine was not detected in mice with the *TauT* ^*-/-*^ deletion. The level of taurine was slightly reduced in the diabetic WT mice, and nearly depleted in the diabetic *TauT* ^*+/-*^ mice. Fig. 1B shows expression of the *p53* tumor suppressor gene (which is activated by hyperglycemia and affects *TauT* transcription) and *TauT* in both non-diabetic and diabetic WT and *TauT* ^*+/-*^ mice. Previous studies have shown that activation of *p53* down-regulates *TauT* expression in renal cells both *in vitro* and *in vivo* (Han 2002 and 2009). The present results confirmed the genotypes of *TauT*-deficient mice used for the current study and the down-regulation of *TauT in vivo* in diabetic mice, particularly in *TauT* ^+/-^ diabetic mice at 5 months after DM induction (Fig. 1C and D).

### General Characteristics of Diabetic Mice

**Fig 2:**
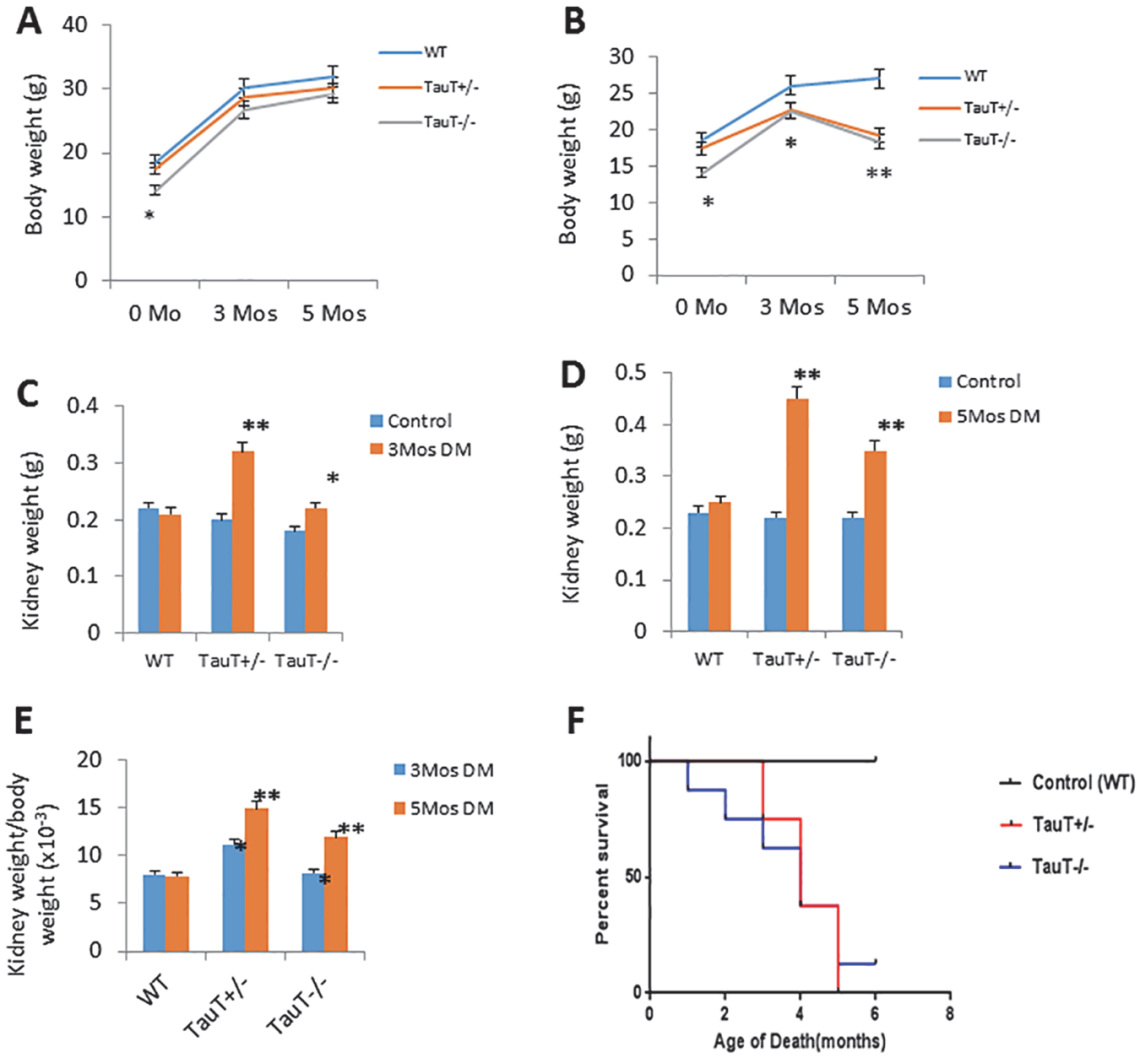
Diabetic *TauT*-deficient mice showed kidney hypertrophy and decreased survival rate. A) Body weight changes in each genotype of control mice; B) Body weight changes in each genotype of diabetic mice; C) Kidney weight changes in each genotype of diabetic mice after 3 months and D) 5 months; E) Kidney weight/body weight ratio in each genotype of diabetic mice after 3 or 5 months; F) Kaplan-Meier plot of survival rate of diabetic mice of each genotype. *p < 0.05 vs controls, **p < 0.01 vs controls.

*TauT* deletion did not alter fasting blood glucose levels in either *TauT* ^+/-^ or *TauT* ^*-/-*^ mice as compared to WT (Table 1). STZ injection induced type 1 diabetes in WT, *TauT* ^+/-^ or *TauT* ^*-/-*^ mice by approximately 12 weeks of age, as determined by elevated fasting blood glucose levels. Three months after diabetes induction, diabetic mice from all genotypes showed significant weight loss (Fig. 2B) as compared to control mice (Fig. 2A), increased urine albumin, and increased BUN levels as compared to controls (Table 1). Interestingly, kidney weight was significantly increased in diabetic *TauT*-deficient mice 3 months after diabetes induction (Fig. 2C). Notably, net kidney weight was nearly doubled in diabetic *TauT* ^+/-^ mice 5 months after diabetes induction (Fig. 2D), while the body weight of these mice was decreased by about 30% compared to controls. The increasing ratio of kidney weight to body weight (Fig. 2E) indicated that progressive renal damage was occurring in diabetic *TauT*-deficient mice, but not in diabetic wild-type mice. Twelve-hour urine volume was increased in diabetic WT and *TauT* ^+/-^ mice, but decreased in diabetic *TauT* ^*-/-*^ mice (Table 1), suggesting possible development of advanced chronic kidney disease. Diabetic nephropathy increased the mortality of *TauT*-deficient mice during the 6-month period of the experiment (Fig. 2F, p<0.001 vs wild-type control). About 50% of diabetic *TauT*-deficient mice died at 4 months, and the remaining mice had to be euthanized because of severe dehydration and inanition.

**Table 1:** General characteristics of wild-type and *TauT*-deficient control and diabetic mice.

	Wild-type		*TauT* ^+/-^		*TauT* ^-/-^	
Characteristic	Control	Diabetic	Control	Diabetic	Control	Diabetic
3 months after induction of DM						
Blood glucose (mg/dL)	119 ± 15	360 ± 58*	125 ± 25	36800B003182*	125 ± 35	385 ±105*
Urine albumin/creatinine (x 10^–1^)	1.8 ± 0.6	5.3 ± 1.3*	1.6 ± 0.4	8.2±1.8**	2.4 ± 0.6	10.6±2.3**
BUN (mg/dL)	12.9± 2.4	15.6 ± 2.1	12.9±3.0	21.6±2.0**	15.2±2.8	22.8 ± 2.1*
5 months after induction of DM						
Blood glucose (mg/dL)	128 ± 15	369 ± 34*	139 ± 36	380 ± 56*	136 ± 32	396 ± 60*
Urine albumin/creatinine (x 10^–1^)	3.5 ± 1.1	8.9 ± 2.8*	4.2 ± 1.2	12.4±2.3**	2.7 ± 1.0	21.2±2.8**
BUN (mg/dL)	12.2± 1.8	20.3 ± 2.8*	12.8±2.1	32.8±3.4**	17.2±2.8	31.7±2.9**
12-h urine (µL)	998 ± 88	1220 ± 200*	958 ± 87	1583±129*	856 ± 83	652 ± 79**

DM, diabetes mellitus; BUN, blood urea nitrogen.

*p < 0.05 vs control

**p < 0.01 vs control

### Morphological Changes in Glomeruli in Diabetic *TauT*-deficient Mice

**Fig 3:**
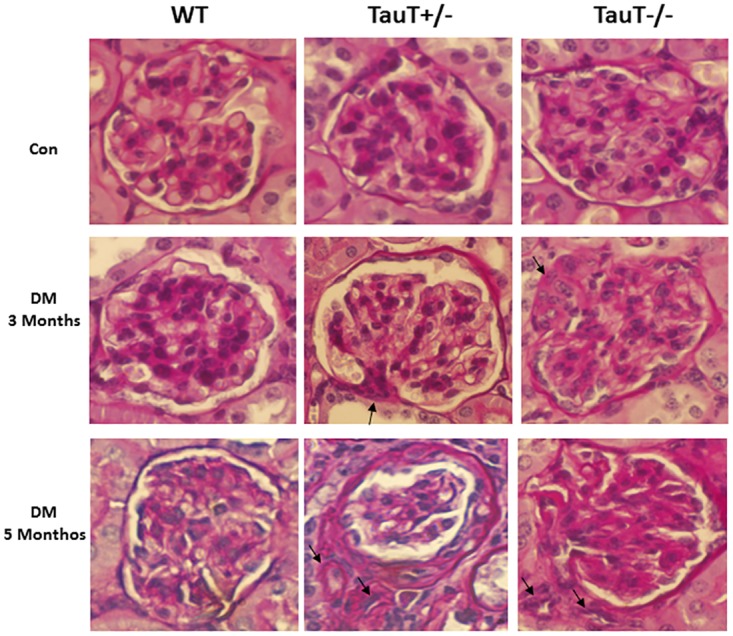
Histology of glomeruli from wild-type (WT) and *TauT*-deficient mice. Arrows show hyalinosis and extension of juxtaglomerular apparatus in diabetic *TauT*+/- and *TauT* ^-/-^ mice at 3 months (middle row). Arrows indicate areas of arteriolar hyalinosis associated with arteriosclerosis, glomerular mesangiolysis and nodular glomerulosclerosis in diabetic *TauT* ^+/-^ and *TauT* ^-/-^ mice at 5 months (bottom row). Hematoxylin and eosin stain; magnification x400. Con, control; STZ, streptozotocin.

As shown in Fig. 3, both WT and *TauT*-deficient diabetic mice developed mesangial expansion 3 months after STZ treatment, a sign of the early stages of diabetic nephropathy; however, it was more prominent in the *TauT* mutant mice, especially in *TauT* ^*-/-*^ mice. Furthermore, nodular lesions and nodular glomerulosclerosis were observed at 5 months after diabetes was induced in *TauT* ^+/-^ and *TauT* ^*-/-*^ mice. The most extensive damage to glomeruli was distributed in the juxtaglomerular area. Arteriosclerosis and hyalinosis of arterioles (arrows) were observed in diabetic *TauT* ^+/-^ and *TauT* ^*-/-*^ mice. It is noteworthy that arteriosclerosis and hyalinosis, when present, often were associated with severe glomerulosclerosis.

### Histologic Renal Lesions in Diabetic *TauT*-deficient Mice

**Fig 4:**
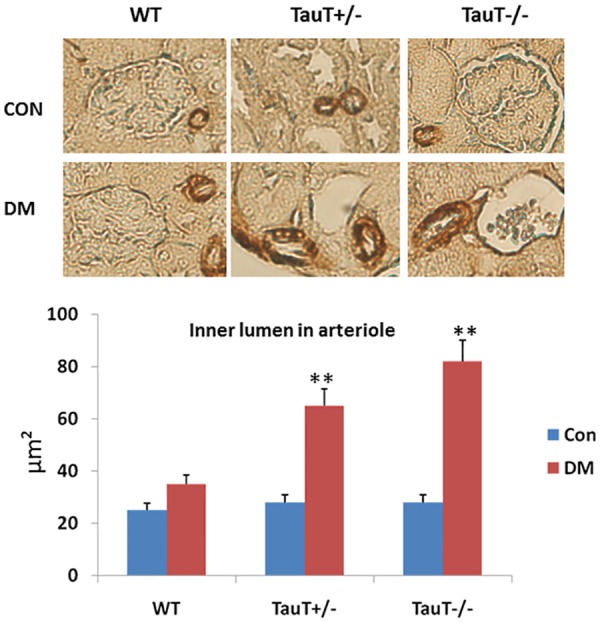
Immunohistochemical analysis of smooth muscle actin (SMA) in arterioles showed thickened and dilated arterioles in *TauT* mutant diabetic mice as compared to wild-type (WT) diabetic mice and non-diabetic controls. The lower panel shows significant differences in the diameter of the inner lumen of arterioles from *TauT* mutant and non-mutant mice. **p < 0.01 vs control. Con, control, DM, diabetes mellitus.

**Fig 5:**
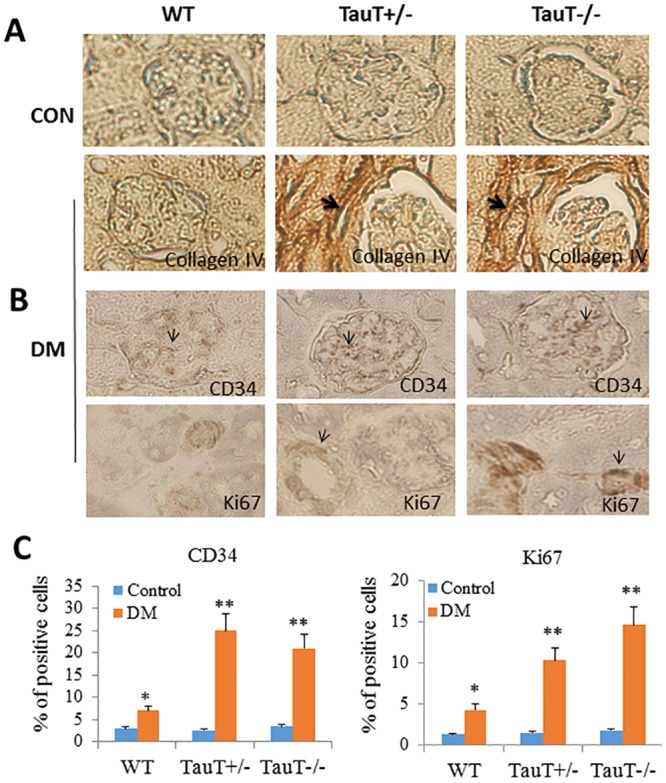
Histologic renal lesions in diabetic *TauT*-deficient mice. A) Immunohistochemical analysis showing deposition of collagen IV in diabetic *TauT* ^+/-^ and *TauT* ^-/-^ mice at 5 months of DM; B) Immunohistochemistry for CD34 and Ki67 (brown color) in diabetic *TauT* ^+/-^ and *TauT* ^-/-^ mice at 5 months of DM; C) Quantitation of CD34- and Ki67-positive cells. *p < 0.05 vs controls, **p < 0.01 vs controls. Magnification: x400.

The most marked changes in the diabetic *TauT*-deficient mice were the increased thickness and dilation of both afferent and efferent arterioles of the glomeruli. These features closely replicate the changes in arterioles observed in advanced renal disease associated with human diabetic nephropathy. Immunohistochemical staining for smooth muscle actin (SMA) showed that the arterioles were significantly thickened and dilated in *TauT* ^+/-^ and *TauT* ^*-/-*^ diabetic mice at 5 months compared to the controls and WT diabetic mice (Fig. 4A and B). These pathological changes were often associated with glomerular necrosis. Although wild-type diabetic mice also demonstrated some glomerular changes, there was no clear damage to the glomerular structure. Immunohistochemical staining demonstrated that collagen IV was heavily deposited in the juxtaglomerular area in *TauT*-deficient diabetic mice and was associated with mesangial expansion (Fig. 5A).

Endothelial morphology was studied by immunostaining of CD34, a marker for endothelial cells. A generalized increase in endothelial cells in the glomeruli was evident in diabetic *TauT*-deficient mice; this was associated with enhanced endothelial cell proliferation in dilated arterioles and determined by staining Ki-67, an endothelial cell proliferation marker (Fig. 5B). Compared to diabetic WT mice, both CD34 and Ki-67 were increased in both classes of *TauT*-deficient diabetic mice (Fig. 5C).

**Fig 6:**
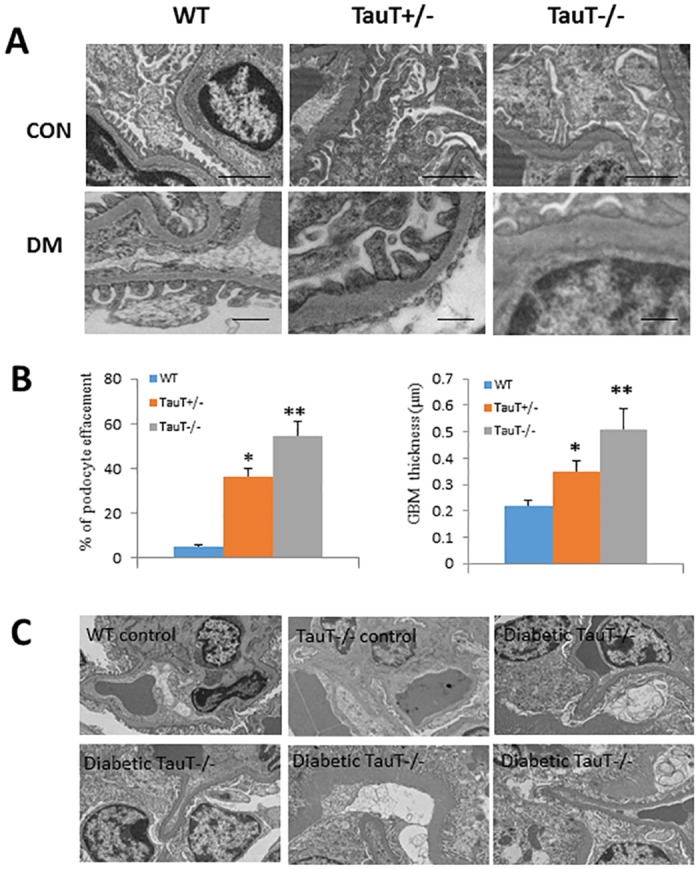
Electron micrographs of glomeruli in wild-type (WT) and *TauT*-deficient diabetic mice. A) Con: lower magnification (5000x) of non-diabetic WT, *TauT* ^+/-^ and *TauT* ^-/-^ mice with preserved podocyte with foot processes and basement membrane (bar = 2 µm). DM: higher magnification (10000x) of diabetic WT kidney with red blood cells in capillary lumen, preserved basement membrane and podocyte with foot processes (bar = 500 nm). B) Percent of podocyte effacement and glomerular basement membrane (GBM) thickness in glomeruli from a diabetic *TauT* ^*+/-*^ mouse and a diabetic *TauT* ^*-/-*^ mouse showing markedly expanded mesangium, thickened GBM, and podocyte effacement in comparison to WT control. C) Diabetic TauT^-/-^ mice showed destruction of capsule membranes as compared to WT and *TauT* ^*-/-*^ non-diabetic mice. *p < 0.05 vs controls, **p < 0.01 vs controls.

Glomerular ultrastructure and glomerular basement membrane thickness in 6-month-old mice was examined by electron microscopy (Fig. 6). Compared with control mice, there was significant (*P* < 0.05) thickening of glomerular basement membrane (GBM) in both diabetic *TauT* ^*+/-*^ mice and diabetic *TauT* ^*-/-*^ mice (Fig. 6A). Severe podocyte effacement was evident in both genotypes of diabetic *TauT*-deficient mice (Fig. 6A and B). Diabetic *TauT* ^*-/-*^ mice showed severe Bowman’s space membrane destruction (Fig. 6C).

Additionally, retinal degeneration was evident in these diabetic *TauT*-deficient mice. Examination of the eyes showed striking retinal changes often characteristic of human diabetic retinopathy, suggesting that the *TauT*-deficient mouse model may also be suitable for studying diabetic retinopathy (data not shown). The broader implications of these findings require a level of investigation and discussion outside the range of the current report and will be addressed in a separate publication.

## Discussion

Diabetic kidney disease, or diabetic nephropathy, is a major cause of morbidity and mortality in people with diabetes. Over the past decade, the quest for an ideal mouse model for diabetes has progressed slowly. Recent studies show that both BRKO-Akita and BTBR *ob/ob* mice develop more advanced diabetic nephropathy than earlier mouse models [10,11]. However, both models have certain limitations, especially with regard to the development of end stage renal disease, as the animals failed to develop severe progressive renal insufficiency. Additionally, the BTBR *ob/ob* mouse model is dependent on leptin deficiency, which is not a characteristic of human diabetes. The lack of reliable animal models that closely mimic human disease has hampered the development of drugs to prevent and/or treat diabetic nephropathy, as well as a deeper understanding of the pathogenesis of this condition.

In this study, we have first demonstrated that both homozygous (*TauT* ^*-/-*^) and heterozygous deletion of the *TauT* gene (*TauT* ^*+/-*^) cause C57BL/6 mice to be highly susceptible to diabetic nephropathy and to display classic characteristics of end-stage renal disease in humans, such as microalbuminuria, azotemia and significant kidney hypertrophy. Morphologic changes include an increase in the mesangial matrix, hyalinosis and fibrous thickening of efferent and afferent arterioles with severe dilation and arteriosclerosis, Kimmelsiel-Wilson nodules, and increasing thickness of glomerular basement membranes, all characteristic of diabetic nephropathy.

Previous studies have shown that dietary taurine restriction results in taurine deficiency (30% decrease) and causes severe renal injury in kittens born of taurine-deficient queens [20]. The bioavailability of taurine is severely reduced in diabetic patients; it is 30% lower than in matched control subjects and similar to the level found in taurine-deficient cats [17,18]. High glucose levels (such as those characteristic of diabetic patients) down-regulate *TauT* expression, possibly through activation of *p53* [21], which transcriptionally down-regulates *TauT* by binding to *TauT* promoter DNA [22]. This can then result in intracellular taurine depletion [23,24]. Unlike humans, rodents, including mice, are able to synthesize sufficient taurine to maintain body stores. But taurine deficiency can be achieved by genetic manipulation of the *TauT* gene, as demonstrated in *TauT* knockout mice. Notably, *TauT* null mice develop clinically relevant age-dependent disorders, including cardiomyopathy, liver disease, and kidney malfunction [19,25,26]. Taurine appears to play an important role in protecting against the effects of stress-induced apoptosis, including diabetic vasculopathy, by inhibition of renal lectin-like oxidized low-density lipoprotein receptor-1 (LOX-1), which facilitates the uptake of oxidized low-density lipoprotein (oxLDL) and induces endothelial dysfunction [27,28]. Taken together, the above evidence strongly suggests that taurine deficiency is a potential key risk factor for chronic kidney disease by contributing to the progression of diabetic nephropathy.

The C57BL/6 strain of mice used as background for these transgenic colonies are a traditional model for diabetic studies, as they exhibit the high blood glucose levels associated with human diabetes. However, although they display many common features of the disease, they are not susceptible to the renal pathologic alterations that are a hallmark of the human diabetic condition and lead to end stage renal disease. Compared to other strains of mice, such as DBA/2, C57BL/6 mice are genetically resistant to STZ-induced diabetic nephropathy, due in part because they express high levels of TauT, as shown in this study. Here, we have shown that *TauT* deficiency predisposes C57BL/6 mice to STZ-induced diabetic nephropathy, as determined by renal physiology, renal histopathology, and electron micrographs of glomeruli of diabetic *TauT*-deficient mice. High glucose levels result in depletion of TauT and taurine in diabetic *TauT* heterozygous deletion mice and cause progressive diabetic nephropathy, which mimics the features of human disease. Lower TauT status is likely a critical risk factor for development of diabetic nephropathy. In diabetic humans, the kidney has a limited ability to reabsorb taurine, leading to taurine deficiency. *TauT*-deficient animals closely mimic all the changes that occur in humans as a result of diabetes, and demonstrate the characteristics of advanced diabetic nephropathy. However, the mechanisms of how heterozygous *TauT* mutant diabetic mice develop progressive nephropathy in a relatively short period of time (5 months after induction of diabetes by STZ) and show similar pathological changes to the kidneys as those observed in homozygous *TauT* mutant diabetic mice need to be investigated further. The *TauT* ^*+/-*^ mouse may serve as an ideal animal model for the study of human diabetic kidney disease, as it is a more accurate representation of the human diabetic condition, in which there is a deficiency but not complete absence of taurine and the taurine transporter. Hence, it may be a more appropriate model to study progressive diabetic kidney disease. Recently, Fiorina and colleagues have identified podocyte B7–1 as a potential therapeutic strategy for the prevention or treatment of diabetic nephropathy [29]. We also found that diabetic *TauT* deficiency mice suffer severe podoyte injury. Hence, examining expression of podocyte B7–1 in diabetic *TauT*-deficient mice may help us to fully understand the role of *TauT* deficiency in diabetic nephropathy.

In conclusion, taurine deficiency appears to be a major risk factor for the development of nephropathy (and perhaps retinopathy) in diabetic patients. Whether the *TauT*-deficient mouse may serve as an ideal model for the study of diabetic nephropathy and therapeutic strategies to slow the progression of chronic kidney disease needs to be determined in future studies.
